# Impact of family planning messages on modern contraceptive use: an observational study of women of reproductive age in Zambia 2007–2018

**DOI:** 10.1136/bmjgh-2026-024080

**Published:** 2026-10-30

**Authors:** Joseph Kazibwe, Jesper Sundewall, Felix Masiye, Mia Kolak, Marie Klingberg-Allvin, Bjorn Ekman

**Affiliations:** 1 Department of Clinical Sciences, Malmö, Lund University, Malmö, Sweden; 2 University of KwaZulu-Natal, Durban, South Africa; 3 Department of Economics, University of Zambia, Lusaka, Zambia; 4 Department of Women and Children’s Health, Karolinska Institutet, Stockholm, Sweden

**Keywords:** Global Health, Health policy, Health education and promotion

## Abstract

**Introduction:**

Reducing unintended pregnancies in low-income and middle-income countries requires enhancing the use of modern contraceptives. Country level evidence on the role of the media and counselling in this process is scanty. The aim of the study is to estimate the impact of family planning messages received through mass media and counselling by health workers on modern contraceptive use among women of reproductive age in Zambia over the study period of 2007 to 2018.

**Methods:**

We analysed three rounds of Zambia Demographic and Health Survey data (2007, 2013/14, 2018). We used different modelling approaches, including logistic regressions, probability weighting and propensity score matching to determine the average treatment effects (ATE) of media messages and counselling by health workers. ATE were also estimated by year. Post-estimation and robustness checks were conducted using overlap plots and bootstrapping.

**Findings:**

Across all surveys, a total of 19 958 women of reproductive age (15–49 years) were identified for inclusion in the study. The magnitude of impact of family planning messages varied by mode of delivery of the messages. Receiving family planning messages through a combination of health worker counselling and mass media had the most significant positive impact on contraceptive use (ATE 17.06%) followed by counselling (ATE 14.55%) and mass media messages only (ATE 3.37%). The impact of the different modes of delivery of family planning varied by year, showing a decreasing trend.

**Conclusion:**

The mode of delivery influences the impact of family planning messages on modern contraceptive use among women. Family planning counselling and combined approaches outperform mass media messages only. A declining impact over time raises concerns and highlights the need for further investigation into delivery effectiveness.

**WHAT IS ALREADY KNOWN ON THIS TOPIC**

**WHAT THIS STUDY ADDS**

- Generally, receiving family planning messages increases the chances of using modern contraception among sexually active women of reproductive age in Zambia.
- The impact of receiving family planning messages on modern contraceptive use varies with the mode of delivery of the messages: receiving family planning messages through a combination of family planning counselling and mass media yielded the most impact, followed by counselling alone and mass media had the smallest impact.
- The impact of the different modes of delivery of family planning decreased over time, indicating a decreasing trend in their impact across the study period.

**HOW THIS STUDY MIGHT AFFECT RESEARCH, PRACTICE OR POLICY**

- Policymakers should consider prioritise delivering family planning messages through both counselling and mass media, as this combined approach shows the greatest potential to increase modern contraceptive use.
- The observed decline in the impact of different message-delivery modes highlights the need for further research to understand the factors driving this trend and to identify strategies for improving future communication efforts.

## Introduction

 Unmet need for contraception is still widespread globally with over 164 million women experiencing unmet need for contraception as of 2022.^1^ Access to evidence-based contraceptive information and modern contraception is essential in enabling women to plan childbirth, space children and avert unintended pregnancies, which consequently contributes to the overall reduction of maternal mortality.^2–4^

The right and ability to plan when to have children through the use of contraception plays a critical role in reducing unintended pregnancies and, more importantly, lowering the proportion of high-risk pregnancies that may result in maternal deaths.^2 5^ Globally, fertility rates have declined considerably from an average of 3.2 live births per woman in 1990 to 2.2 in 2024.^6^ During the same period, the use of modern contraceptives among women of reproductive age increased from 42% in 1990 to 49% in 2019.^7^ This rise in contraceptive use has been linked to improvements in maternal health outcomes, including reduction in maternal mortality.^3 5 8^

Despite the progress, Sub-Saharan Africa (SSA) continues to experience a high total fertility rate averaging 4.6 children per woman as of 2021,^9 10^ almost double the global average. This elevated fertility rate is partly driven by the high incidence of unintended pregnancies, which account for 29% to 54% of all pregnancies in the region. Unintended pregnancies are particularly prevalent among married women (occurring six times more frequently than among unmarried women) and among adolescents aged 15 to 19.^11^ These pregnancies are associated with adverse maternal outcomes, including low birth weight, neonatal mortality and maternal mortality.^12^ The high total fertility rate and widespread unintended pregnancies in SSA may explain why the maternal mortality ratio (MMR) in SSA is also still high (542 maternal deaths per 100 000 live births)^13^ compared with the global average (197 maternal deaths per 100 000 live births).^14^ SSA accounts for approximately 69% of all maternal deaths globally.^15^

The use of modern contraception is important for reducing maternal mortality and unintended pregnancies. The benefits of modern contraception may not be fully realised due to persistent misconceptions and misinformation about modern contraceptive use^16 17^ and health information inequality.^18^ Family planning messages, whether delivered by health workers or through the media, can help counter misinformation, increase knowledge of contraceptive methods and their benefits for both men^19^ and women.^20^ Family planning messages also allow the population to overcome misconceptions about modern contraception;^21^ however, this could be dependent on quality of care^22 23^ and media information.^20^

Investigating how family planning messages are delivered is crucial, as the mode of delivery can influence their effectiveness. Family planning counselling, for example, can vary by timing, structure and context, such as antenatal, postnatal, pre-abortion, group or individual counselling. These variations may have distinct effects on contraceptive uptake^24^ depending on the quality and relevance of the counselling provided.^25^ For mass media, family planning messages can be delivered via radio, television and newspapers. All these may have different influence on modern contraceptive use and may differ by context.^26 27^ Radio-based family planning messages have shown a stronger influence on modern contraceptive use compared with television-based messages^28^ contrary to the findings in Asia.^26 27^

Previous studies have examined the impact of family planning messages on modern contraceptive use. For example, Dwomoh *et al*^29^ evaluated access to family planning messaging on contraceptive use in the SSA. However, most existing research has focused primarily on mass media channels and has not systematically compared different modes of delivery. Evidence on the effectiveness of family planning counselling is mixed,^24^ and a recent systematic review concluded that results remain inconclusive.^30^ Overall, there is a lack of studies that directly estimate and compare the impact of different modes of delivery of family planning messages on modern contraceptive use.

Zambia is a relevant case, with persistently high levels of maternal mortality and low use of modern contraceptives, especially in rural areas and among women with lower socioeconomic status.^31 32^ To increase contraceptive uptake, the Zambian government has implemented various strategies, including mass media campaigns, outreach activities and the use of community health workers. The limited evidence from Zambia suggests that family planning messages through mass media have increased chances for modern contraceptive use in Zambia.^33 34^ However, these studies are narrow in scope and focus on specific programme areas^34^ or subgroups.^33^

This study contributes to the current evidence on family planning messages by estimating the impact of different modes of delivering family planning messages (through mass media and counselling by health workers) on current use of modern contraception among women of reproductive age in a low-income setting. Specifically, we examine the impact of family planning messages delivered through three channels: counselling by health workers, mass media and a combination of these two modes of delivery. The impact of the modes of delivery was estimated using logistic regression and causal inference techniques. The findings are intended to inform interventions and national policies to increase modern contraceptive use in Zambia and similar contexts.

## Methods

This quasi-experimental study used repeated nationally representative cross-sectional demographic health survey (DHS) data^35^ to estimate the average treatment effects (ATE) of family planning messages through mass media and counselling on modern contraceptive use.

### Setting and study participants

The study uses data from Zambia, a low-income country located in Southern Africa with an estimated population of 20 million as of 2022, of which 50% are female.^36^ The majority of the population (55%) resides in rural areas.^36^ Unmet need for contraception remains high: 16% among married women and 34% among sexually active unmarried women.^31^ All 10 provinces of Zambia are included in this study.

The study used three rounds of the Zambia demographic and health survey (ZDHS) data of 2007, 2013/14 and 2018 (dataset).^35 37^ Combining multiple surveys increased the sample size, improved statistical power, reduced bias and accounted for temporal variation in treatment effects. The study population consisted of sexually active women of reproductive age (15–49 years). Women were defined as sexually active if they had engaged in sexual intercourse within 30 days prior to the interview. This group was selected because they are at the highest risk of unintended pregnancies compared with women who were not sexually active.

### Variables

To investigate the impact, the study outcome variable is modern contraceptive use. Modern contraceptive use is the current use of any of the modern types of contraception including both short-term and long-term methods, for example, condoms, sterilisation, injectables, intrauterine devices, implants and pills. Modern contraceptive use variable is a dichotomous categorical variable coded as 1 for yes (currently using modern contraception) and 0 for no (currently not using modern contraception).

This study considers three main methods (exposure variables) through which the participants received family planning messages.

1. Mass media: access to family planning messages through only mass media (through either radio, TV or newspapers); referred to as ‘mass media’ hereafter.
2. Family planning counselling: access to family planning messages only during family planning counselling by either health worker at health facility or field health worker within the community; referred to as ‘family planning counselling’ hereafter.
3. Both family planning counselling and mass media: access to family planning messages through both mass media and family planning counselling by either health worker at health facility or field worker within the community; referred to as ‘both family planning counselling and mass media’ hereafter.

Family planning counselling by health worker at health facility involves women who received family planning messages through family planning counselling by a health worker at the health facility within the last 12 months prior to the date of the interview. The health workers are usually nurses or midwives. A health facility includes any facility that provides health services recognised as a part of the health system structure.

Family planning counselling by field health worker entails women who received family planning messages through family planning counselling at their respective homes/households/community provided by a field health worker within the last 12 months prior to the date of the interview. The health worker in this case is usually a community volunteer or field health worker.

Figure 1 shows the frequencies of the different modes of delivery of family planning messages (exposure variables) considered in this study. All exposure variables are categorical.

**Figure 1:**
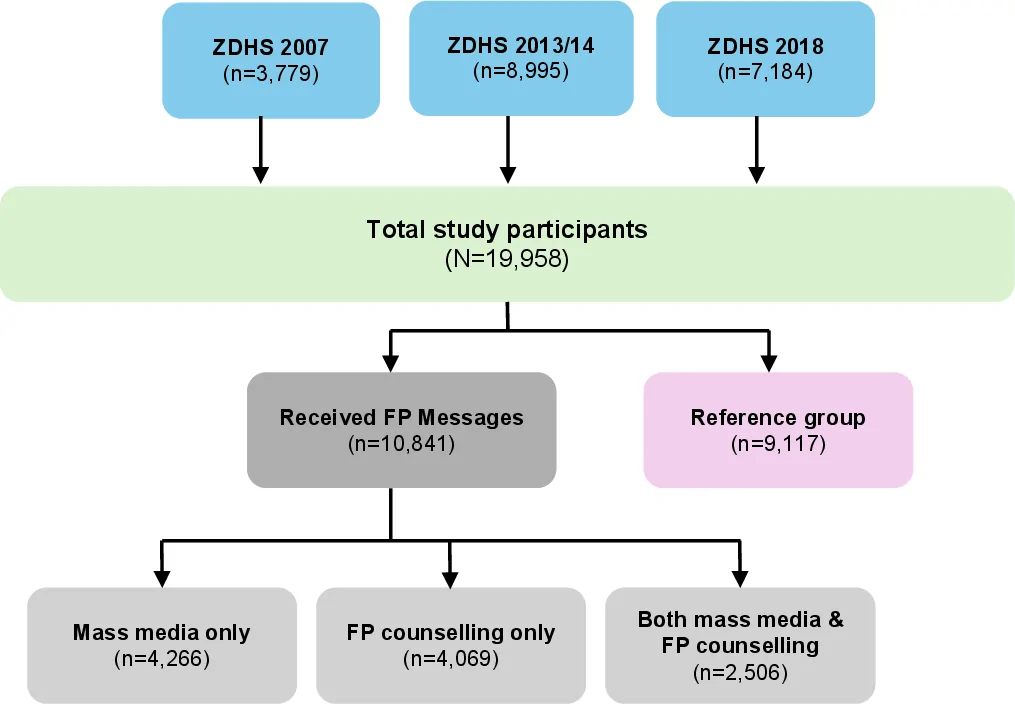
Different modes of delivery of family planning messages and the number of participants that received the messages through each mode. FP, family planning; ZDHS, Zambia demographic and health survey.

The independent variables are demographic characteristics of the study participants that is age group, wealth status, highest level of education attained, religion, employment status, parity, sex of household head, insurance coverage, year of data collection and being in a union. A household head or head of the household is the member of the household that the respondent perceives to be responsible for the household.^38^ This could be influenced by the culture, income or age of the household members.^38^ Being in a union is staying with a partner whether married or not.

The three datasets ZDHS 2007, 2013/14 and 2018 were appended to create one combined dataset with year of data collection as a dummy variable. The total number of sexually active women of reproductive age was 19 958 (figure 1).

### Statistical analyses

We assessed the impact of the modes of delivery of family planning messages on the use of modern contraceptives using survey-weighted logistic regression models, adjusting for potential confounding variables as our foundational model. At this stage, three models were analysed, first, the impact of family planning messages delivered through mass media on modern contraceptive use, second, the impact of messages delivered through family planning counselling and lastly, the impact of messages delivered through both mass media and counselling. However, there is a potential problem of self-selection, making the treatment variables endogenous. The models are thus prone to selection bias and unobservable confounding. Therefore, to address any potential heterogeneity and selection bias, we estimated the ATE of modes of delivery of family planning messages on modern contraceptive use using three causal inference techniques: augmented inverse probability weighting (AIPW),^39^ inverse probability weighting (IPW)^40^ and propensity score matching (PSM).^41^ These estimators allowed for a comparison between individuals that received family planning messages through a specific mode and those that did not receive any family planning messages. The AIPW estimator has both regression adjustment and IPW and offers double robustness; thus, it yields consistent estimates of the ATE if either the treatment assignment model (propensity score) or the outcome model is correctly specified.^39^ To validate the robustness of the AIPW estimates, we supplemented the analysis with IPW and PSM approaches.

To understand the trend of impact of the modes of delivery of family planning messages, we run estimated the ATE of each mode of delivery by year.

We conducted post-estimation checks to assess the validity of the causal inference assumptions. This included evaluating the overlap assumption using graphical diagnostics (eg, overlap plots) and inspecting the distribution of covariates across treatment groups. Robustness of the treatment effect estimates was assessed by comparing results across the multiple estimators (AIPW, IPW and PSM) and implementing bootstrap resampling with 100 iterations to evaluate the stability of standard errors and CIs. The significance of the findings was based on 95% CIs of the estimates. All analyses were carried out using Stata 18.

### Patient and public involvement

No patient was involved in this study. The findings of this study will be presented the Ministry of Health of Zambia for consideration and action.

## Results

### Study participant characteristics

The study included 19 958 participants, all women of reproductive age (15–49 years). Majority of the participants included were from Eastern province (13.23%) followed by Lusaka province (11.61%), Southern province (11.11%) and Copperbelt province (10.25%). The average age of the participants was 30.7 years with a minimum of 15 and maximum of 49 years. Most participants were between 20 and 34 years old (58.56%). Over half of all participants (53.62%) had primary education as the highest education level attained. Majority of the participants (89.69%) are in a union (stay with a partner). Household heads are majorly male (86.67%). The largest proportion of participants reside in rural areas (60.13%). Table 1 below shows the demographics of the study participants combined from DHS 2007, 2013/14 and 2018.

**Table 1:** Characteristics of study participants

Variable	Control group		Mass media		FP counselling		Both FP counselling & mass media		Total	
	N	%	N	%	N	%	N	%	N	%
Province										
Central	960	10.53	412	9.66	302	7.42	214	8.54	1888	9.46
Copperbelt	892	9.78	594	13.92	289	7.1	271	10.81	2046	10.25
Eastern	940	10.31	483	11.32	680	16.71	537	21.43	2640	13.23
Luapula	904	9.92	386	9.05	440	10.81	234	9.34	1964	9.84
Lusaka	1012	11.1	622	14.58	381	9.36	302	12.05	2317	11.61
Muchinga	890	9.76	377	8.84	411	10.1	190	7.58	1868	9.36
Northern	1001	10.98	336	7.88	381	9.36	225	8.98	1943	9.74
North western	834	9.15	469	10.99	498	10.03	184	7.34	1895	9.49
Southern	1146	12.57	392	9.19	441	10.84	238	9.5	2217	11.11
Western	538	5.9	195	4.57	336	8.26	111	4.43	1180	5.91
Total	9117	100	4266	100	4069	100	2506	100	19 958	100
Age group										
15–19	1013	11.11	258	6.05	300	7.37	143	5.71	1714	8.59
20–24	1675	18.37	692	16.22	931	22.88	488	19.47	3786	18.97
25–29	1786	19.59	907	21.26	919	22.59	605	24.14	4217	21.13
30–34	1519	16.66	826	19.36	763	18.75	577	23.02	3685	18.46
35–39	1303	14.29	663	15.54	626	15.38	361	14.41	2953	14.8
40–44	1001	10.98	531	12.45	343	8.43	216	8.62	2091	10.48
45–49	820	8.99	389	9.12	187	4.6	116	4.63	1512	7.58
Total	9117	100	4266	100	4069	100	2506	100	19 958	100
Wealth index										
Poorest	2183	23.94	295	6.92	1149	28.24	268	10.69	3895	19.52
Poorer	2093	22.96	551	12.92	1060	26.05	406	16.2	4110	20.59
Middle	2087	22.89	791	18.54	936	23	519	20.71	4333	21.71
Richer	1717	18.83	1173	27.5	601	14.77	630	25.14	4121	20.65
Richest	1037	11.37	1456	34.13	323	7.94	683	27.25	3499	17.53
Total	9117	100	4266	100	4069	100	2506	100	19 958	100
Highest level of education attained										
No education	1189	13.04	238	5.58	467	11.48	144	5.75	2038	10.21
Primary	5300	58.13	1789	41.94	2516	61.83	1096	43.74	10 701	53.62
Secondary	2385	26.16	1747	40.95	1013	24.9	1029	41.06	6174	30.94
Higher	240	2.63	490	11.49	73	1.79	235	9.38	1038	5.2
Missing	3	0.03	2	0.05	0	0	2	0.08	7	0.04
Total	9114	100	4264	100	4069	100	2504	100	19 951	100
Religion										
Catholic	1542	16.91	799	18.73	657	16.15	444	17.72	3442	17.25
Protestant	7406	81.23	3419	80.15	3352	82.38	2035	81.21	16 212	81.23
Muslim	43	0.47	16	0.38	26	0.64	11	0.44	96	0.48
Other	117	1.28	25	0.59	24	0.59	10	0.4	176	0.88
Missing	9	0.1	7	0.16	10	0.25	6	0.24	32	0.16
Total	9110	100	4266	100	4069	100	2506	100	19 958	100
Union										
No	1065	11.68	492	11.53	338	8.31	162	6.46	2057	10.31
**Yes**	8052	88.32	3774	88.32	3731	91.69	2344	93.54	17 901	89.69
**Total**	9117	100	4266	100	4069	100	2506	100	19 958	100
Currently working										
No	4457	48.89	1722	40.37	1795	44.11	1015	40.5	8989	45.04
Yes	4630	50.78	2540	59.54	2270	55.79	1486	59.3	10 926	54.75
Missing	30	0.33	4	0.09	4	0.1	5	0.2	43	0.22
Total	9117	100	4266	100	4069	100	2506	100	19 958	100
Sex of household head										
Male	7872	86.34	3676	86.17	3533	86.83	2217	88.47	17 298	86.67
Female	1245	13.66	590	13.83	536	13.17	289	11.53	2660	13.33
Total	9117	100	4266	100	4069	100	2506	100	19 958	100
Place of residence										
Urban	3085	33.84	2502	58.65	1092	26.84	1279	51.04	7958	39.87
Rural	6032	66.16	1764	41.35	2977	73.16	1227	48.96	12 000	60.13
Total	9117	100	4266	100	4069	100	2506	100	19 958	100
Parity										
No child	956	10.49	465	10.9	122	3	95	3.79	1638	8.21
One child	1265	13.88	579	13.57	603	14.82	393	15.68	2840	14.23
Two children	1361	14.93	705	16.53	652	16.02	442	17.64	3160	16
Three children	1240	13.6	646	15	581	14	406	16.2	2873	14.4
Four children	1068	11.71	524	12.28	554	13.62	355	14.17	2501	12.53
More than five children	3227	35.4	1347	31.58	1557	38.26	815	32.52	6946	34.8
Total	9117	100	4266	100	4069	100	2506	100	19 958	100
Year of DHS										
2007	1607	17.63	1138	26.68	511	12.56	523	20.87	3779	18.93
2013/14	3922	43.02	2193	51.41	1606	39.47	1274	50.84	8995	45.07
2018	3588	39.36	935	21.92	1952	47.97	709	28.29	7184	36
Total	9117	100	4266	100	4069	100	2506	100	19 958	100

Household head is a person considered responsible for the household and self identifies as such. S/he can be designated based on economic status, age or sex.

DHS, demographic and health survey; FP, family planning.

A total of 10 841 participants reported they had accessed family planning messages through one of the three modes of delivery while 9117 participants had not received any family planning messages. The proportion of individuals receiving family planning messages through mass media increases with increasing wealth index while the proportion of those receiving family planning messages through counselling decreases with increasing wealth index. The ORs of receiving family planning messages by wealth quintile are available in online supplemental material 1. Participants in the poorest quintile are more likely to receive counselling compared with those in higher quintiles while the reverse is true for receiving family planning messages through mass media and both mass media and counselling.

### Trend of proportions for modes of delivery of family planning messages

The share of study participants receiving family planning messages through counselling increased gradually from 13.5% in 2007 to 27.2% in 2018. The share of individuals not receiving any family planning messages also increased from 42.5% in 2007 to 49.9% in 2018.

The biggest share of individuals (>40%) across the years did not receive any family planning messages through any of the modes of delivery. However, there was a decreasing trend for mass media and both counselling and mass media (figure 2).

**Figure 2:**
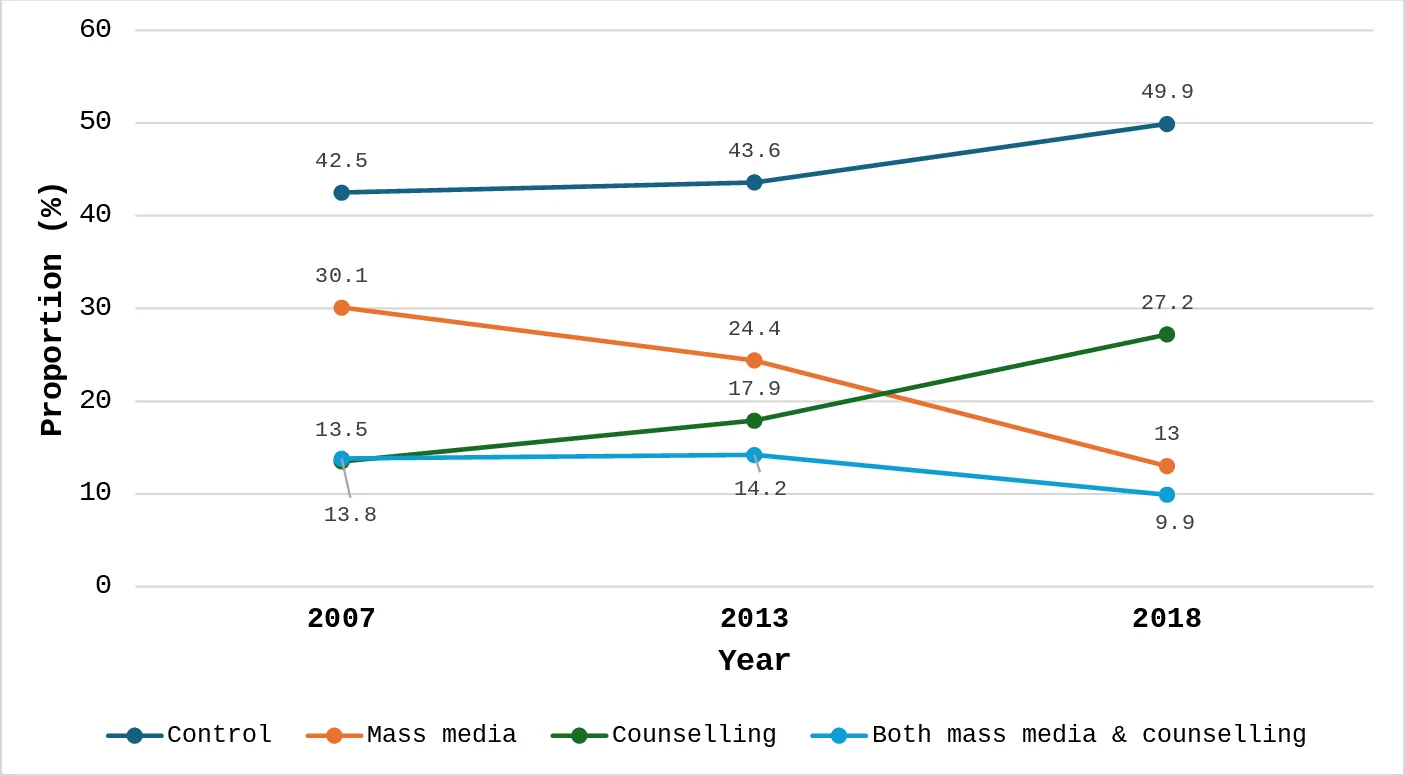
Proportion of people that received family planning messages through each method of delivery by year.

### Impact of mode of delivery of family planning messages on the utilisation of modern contraceptives based on the logistic regression and causal inference techniques

Logistic regression results showed that family planning messages by all three modes of delivery significantly increased the odds of modern contraceptive use, both before and after adjusting for relevant background characteristics, compared with not receiving any family planning messages (online supplemental material 2). Among the three modes of delivery of family planning messages, receiving messages through both mass media and counselling had the highest likelihood of modern contraceptive use (adjusted OR (AOR) 1.73), followed by counselling alone (AOR 1.47) and mass media alone (AOR 1.18). Based on the beta coefficients (table 2), the ranking of effect sizes by delivery mode was consistent with that of the ORs. Messages delivered through mass media had the smallest coefficient (0.17), followed by those delivered through counselling alone (0.38). Messages delivered through both mass media and counselling had the largest coefficient (0.55).

**Table 2:** Impact of modes of delivery of family planning messages on modern contraceptive use using different ATE estimators

Mode of delivery of FP messages	N	Logistic regression			Augmented inverse probability weighting (AIPW)				Inverse probability weighting (IPW)			Propensity score matching (PSM)		
		Coefficient	95% CI		Coefficient (ATE)	95% CI			Coefficient (ATE)	95% CI		Coefficient (ATE)	95% CI	
			Lower bound	Upper bound		P value	Lower bound	Upper bound		Lower bound	Upper bound		Lower bound	Upper bound
Mass media														
Mass media only	13 306	0.1665***	0.063	0.271	0.0337***	0.001	0.0130	0.0544	0.0333**	0.013	0.054	0.0402***	0.018	0.063
Counselling														
FP counselling only	13 113	0.3844***	0.270	0.499	0.1455***	0.000	0.1272	0.1639	0.1475***	0.129	0.166	0.1403***	0.119	0.161
Combination of both														
FP counselling and mass media	11 553	0.5485***	0.435	0.662	0.1706***	0.000	0.1457	0.1954	0.1716***	0.146	0.197	0.1673***	0.138	0.197

*P value ≤0.05, **p value ≤0.01, ***p value ≤0.001.

ATE, average treatment effects; FP, family planning.

Table 2 presents the logistic regression coefficients and estimated ATE of different modes of delivery of family planning messages on modern contraceptive use, using AIPW estimator and other estimators IPW and PSM. All three delivery modalities, mass media, family planning counselling and a combination of both, significantly increased modern contraceptive use. For the ATE, the greatest estimate was observed among individuals exposed to both counselling and mass media, with an estimated increase of 17.06 percentage points in modern contraceptive use. Receiving FP messages through counselling alone resulted in a 14.55 percentage point increase, while through mass media alone had the smallest ATE, increasing modern contraceptive use by 3.37 percentage points. The ATE results from IPW and PSM are similar to those of AIPW in terms of magnitude, direction and significance of impact. Therefore, the results are robust and reliable, insensitive to the application of different estimators. Across wealth-status subgroups, the impact of receiving family planning messages through the different delivery modes was greatest among women in the poorest wealth quintile and smallest among those in the richest wealth quintile (online supplemental material 3).

### Post estimation and robustness checks

To assess the validity of the overlap assumption required for the AIPW estimator, we examined propensity score distributions using graphical overlap diagnostics (online supplemental material 4). The plots demonstrated sufficient common support (overlap) across treatment groups, indicating that the overlap condition was generally satisfied. The bootstrapping results (online supplemental material 5) confirmed that the standard errors were consistent across resamples, supporting the robustness of our inference.

### Trend of the impact by year

Receiving family planning messages through counselling and both counselling and mass media significantly increased modern contraceptive use over the years while messages received through mass media significantly increased modern contraceptive use in 2007 and 2013 but not 2018. The impact of receiving family planning messages through counselling declined from 16.8% in 2007 to 13.4% in 2018, while the impact of receiving messages through both counselling and mass media decreased from 19.8% in 2007 to 11.8% in 2018. The impact of the messages received through each of the three delivery modes shows a decreasing trend over the years to the extent of becoming negative for those received through mass media although not significant (table 3).

**Table 3:** Trend of the impact of different modes of delivery of family planning messages by year estimated by AIPW

Mode of delivery of FP messages	2007			2013			2018		
	ATE	Lower bound	Upper bound	ATE	Lower bound	Upper bound	ATE	Lower bound	Upper bound
Mass media	0.075***	0.036	0.113	0.043***	0.016	0.072	−0.011	−0.052	0.03
Family planning counselling	0.168***	0.12	0.217	0.146***	0.117	0.175	0.134***	0.107	0.162
Both mass media and counselling	0.198***	0.141	0.256	0.188***	0.154	0.222	0.118***	0.074	0.162

*P value ≤0.05, **p value ≤0.01, ***p value ≤0.001.

AIPW, augmented inverse probability weighting; ATE, average treatment effects; FP, family planning.

## Discussion

We find that receiving family planning messages has a positive significant impact on modern contraceptive use among sexually active women in Zambia. The greatest impact was observed among individuals that received messages through both counselling and mass media, followed by counselling alone, and lastly, those receiving messages through only mass media. However, the impact of the messages received through these modes of delivery shows a decreasing trend over the years.

The impact of family planning messages on modern contraceptive use varies in magnitude depending on the mode of delivery. Messages disseminated through traditional mass media channels, such as radio, television and newspapers, had modest increases in modern contraceptive use.^20 29 30 42 43^ Our analysis found that exposure to mass media alone led to an approximate three-percentage point increase in modern contraceptive use, consistent with findings from Babalola *et al*^42^ and Glennerster *et al*^20^ conducted in SSA countries. However, other studies in SSA have reported limited or no impact of mass media messages among specific population subgroups, such as married adolescents in Senegal and Burkina Faso,^44^ and rural residents in Ethiopia.^43^ These inconsistencies likely reflect contextual factors, including cultural norms, educational levels and trust in information sources.^29^

In contrast, family planning counselling showed a more substantial positive impact on modern contraceptive use. This aligns with evidence from a systematic review by Cavallaro *et al*^24^ and other recent studies.^25 45–48^ However, it should be noted that the effectiveness of counselling could depend on its quality,^25^ timing (eg, antenatal or postnatal)^49^ and integration with other health services.^49^

Importantly, receiving family planning messages through a combination of mass media and counselling yielded the greatest impact. This suggests a synergistic effect, where broad-reaching media campaigns complement the personalised nature of counselling to enhance contraceptive use.^27^ Integrating the use of mass media and counselling deliver family planning messages improves both awareness and understanding, supporting informed decision-making and sustained contraceptive use.^50^ Such integration may be especially beneficial in LMICs like Zambia, where barriers to modern contraceptive use often stem from both informational gaps and cultural constraints.^51^

The magnitude of impact observed for family planning messages delivered through counselling was greater than the messages received through mass media. This difference may be attributed to the interactive nature of counselling which allows an individual to engage directly with the health providers with opportunity to ask questions if anything is unclear and receive tailored information.^52^ Family planning counselling sessions usually involve a needs assessment, discussion of available contraceptive options and a shared decision-making process.^53^ This personalised approach helps women understand their contraceptive needs and select methods that best suit their circumstances.^52 54^

Additionally, clearly communicating potential side effects during counselling has been shown to increase both the uptake of new methods and the continued use of existing ones.^24^ Counselling also nurtures a sense of autonomy, empowering women, including adolescents and other vulnerable groups, to make informed and confident decisions about their reproductive health.^55^ These features likely contribute to the stronger impact of counselling on modern contraceptive use.^54^

In contrast, mass media communication is generally one-directional, offering limited opportunities for clarification or personalised support. This can hinder its effectiveness, especially when individuals face contextual barriers or hold misconceptions.^56^ Additionally, the content and framing of media messages may affect their influence, potentially leading to scepticism or misinterpretation.^20^ These dynamics help explain why counselling tends to yield a greater impact on contraceptive uptake.

Our study revealed a declining trend in the effectiveness of all three modes of delivering family planning messages over time. While previous research has documented the waning influence of mass media in promoting modern contraceptive use in SSA,^42^ this decline has not been observed for family planning counselling. The trend may reflect evolving media consumption patterns, particularly among younger populations who increasingly rely on digital platforms for health information.^57 58^ As mobile internet and social media access expand, traditional media channels appear to be losing their reach, reflected in the decreasing usage proportions observed in this study, thereby reducing their impact on reproductive health behaviours. Furthermore, the growing reliance on social media may have amplified the spread of misinformation about modern contraceptive use.^59^ Studies have shown that social media platforms can contribute significantly to the dissemination of inaccurate health information.^60 61^ These dynamics highlight the urgent need for high-quality, targeted family planning messages that are both credible and contextually relevant, to effectively counter misinformation and support informed reproductive health decisions. Although the reasons for the declining effectiveness of the modes of delivery of family planning messages on modern contraception remain unclear, there are several plausible explanations that can be considered. First, changes in the quality, content or person-centredness of counselling services may have diminished the impact of counselling-based interventions over time. Second, increasing workload and competing demands on healthcare providers may have reduced the time available for effective counselling. Third, evolving social norms, fertility preferences and contraceptive attitudes may have altered how women perceive and respond to family planning messages. Finally, shifts in the demographic and socioeconomic characteristics of women reached through different communication channels may have influenced the effectiveness of the modes of delivery over time. These hypotheses require further investigation through qualitative and mixed-methods research. In particular, qualitative research is needed to understand the factors underlying the observed decline in the effectiveness of different modes of delivery of family planning messages on modern contraceptive use over time. Findings from such research could inform the development of plausible hypotheses to explain the observed trend.

Further, an upward trend in the proportion of women not receiving family planning messages over time is a notable and concerning finding. This pattern suggests potential limitations in the reach and effectiveness of existing communication channels and delivery mechanisms. The observed increase warrants further investigation to identify underlying factors contributing to this trend, including possible disparities in access, geographic or socioeconomic barriers, and shifts in programmatic implementation.

This study highlights the importance of applying an equity lens to the delivery of family planning messages. A substantial proportion of the population, particularly individuals in the lower wealth quintiles, remains unreached by any form of family planning communication. The analysis reveals a socioeconomic gradient in access: mass media-based messaging is more commonly accessed by individuals in higher wealth quintiles, whereas family planning counselling is more prevalent among those in lower socioeconomic groups. Furthermore, the magnitude of impact of the modes of delivery of family planning messages is more profound among people of lower wealth status compared with those of higher wealth status. Given the ability of family planning counselling to reach underserved populations, counselling presents a valuable opportunity for scaling up interventions aimed at increasing modern contraceptive use among disadvantaged communities. Targeting such equity-enhancing strategies is essential for addressing persistent gaps in reproductive health access and outcomes.

### Study implications

This study provides important insights into how the effectiveness of family planning messages varies by mode of delivery, offering a potential explanation for the mixed findings reported in previous literature. Two systematic reviews and meta-analyses reached conflicting conclusions: Rogers *et al* reported a significant positive pooled effect of exposure to family planning messages, while Safieh *et al* found no overall association, despite most included studies showing a positive direction of effect. Notably, both reviews focused primarily on mass media-based messaging, which our study found to have the least impact when compared with counselling or combined exposure. Crucially, neither review accounted for the role of family planning counselling, a mode of communication that, according to our findings, has a substantially greater influence on modern contraceptive uptake. These differences in mode of delivery of the messages may help explain the divergent conclusions in the literature and highlight the need to disaggregate communication channels when evaluating the effectiveness of family planning interventions.

Based on these findings, countries aiming to increase contraceptive use should prioritise the expansion of quality family planning counselling services, particularly in underserved settings. Counselling can empower women with knowledge and support to make informed reproductive health decisions. Future research should go beyond aggregated categories to explore which specific forms of media (eg, radio vs television) and counselling (eg, antenatal vs postnatal) are most effective. The observed decline in effectiveness across all delivery modes is concerning and warrants further investigation, especially regarding counselling, to guide the design of future interventions. Finally, although the combination of family planning counselling and mass media messages appears to be the most effective approach for increasing modern contraceptive use, particularly among women in the poorest wealth quintiles, it is likely to be more costly per beneficiary than either counselling or mass media alone. Therefore, future research should assess the cost-effectiveness of the different delivery modes. Such analyses would account for the relative costs and benefits, including the opportunity costs, of each approach and provide evidence on the most efficient and optimal strategy for delivering family planning messages. This evidence would enable policymakers to make informed decisions about resource allocation, better estimate the resources required for programme implementation and promote the sustainable expansion of family planning interventions.

### Limitations

First, the study focuses exclusively on the mode of delivery of family planning messages and does not account for the content or quality of the messages received. While message content likely influences individual interpretation and behavioural response, its exclusion was necessary given the scope of the available data and does not compromise the validity of the findings. Second, the analysis was restricted to complete cases, excluding participants with missing data (less than 0.25% of all participants had a missing value). Although this approach may introduce bias, we assumed that the data were missing completely at random, minimising the potential impact on the results. Third, being sexually active was defined as having had sex within 30 days prior to the interview date. This definition is limiting and excludes women at risk of pregnancy that had had sex before the 30 days and those that have partners but had not had sex in the last 30 days because the partners were away for example women that have partners who travel for work. This may affect the generalisability of the results. Fourth, although we adjusted for a range of observed characteristics and applied causal inference methods, the study remains susceptible to confounding by indication and residual confounding arising from unmeasured or unobserved factors. For example, women who seek or receive family planning counselling may already be more inclined to use modern contraception than those who do not, potentially biasing the estimated ATE of counselling. In addition, unobserved factors such as intrinsic motivation, self-efficacy, partner support and attitudes toward contraception may have influenced both exposure to family planning messages and contraceptive use, resulting in residual confounding. Lastly, we had aimed to include the latest survey data (DHS 2024); however, this was impossible due to the funding cuts to the US Agency for International Development (USAID) by the US government that paused the publication of the DHS 2024 dataset. This study therefore misses the insights of the current impact of the modes of delivery of family planning messages on modern contraception.

## Conclusion

This study provides evidence that the impact of family planning messages on modern contraceptive use among women in Zambia varies considerably by mode of delivery of the messages. Messages delivered through counselling had a greater effect than those disseminated via mass media, while the combination of both methods yielded the highest impact, suggesting a synergistic benefit of integrating informational and interpersonal communication channels. However, we reveal a socioeconomic divide in access to messages through the modes of delivery. These findings highlight the importance of tailoring family planning interventions to the communication preferences and contextual realities of target populations is essential for maximising impact. Furthermore, the observed decline in the impact of all delivery modes and the increasing proportion of women not receiving any family planning message over time raises critical questions about the evolving dynamics of health communication and the need for adaptive, evidence-based approaches. Continued research is warranted to explore the underlying causes of this trend and to identify innovative strategies for enhancing the reach and effectiveness of family planning messaging.

## Supplementary material

10.1136/bmjgh-2026-024080 online supplemental file 1

10.1136/bmjgh-2026-024080 online supplemental file 2

10.1136/bmjgh-2026-024080 online supplemental file 3

10.1136/bmjgh-2026-024080 online supplemental file 4

10.1136/bmjgh-2026-024080 online supplemental file 5

10.1136/bmjgh-2026-024080 online supplemental file 6

## Data Availability

Data are available in a public, open access repository.
