# Supplementary material for: Impact of family planning messages on modern contraceptive use: an observational study of women of reproductive age in Zambia 2007–2018
Source: BMJ Glob Health. 2026 Oct 30;11(10):e024080. doi: 10.1136/bmjgh-2026-024080 (PMC13723780; doi:10.1136/bmjgh-2026-024080)
Supplement: Supplementary file 1 [file bmjgh-11-10-s001.docx]

**Supplementary material 1:** Odds ratios of receiving family planning messages through the different methods across wealth quintiles.

| **Wealth quintile** | **Mass media** | **Counselling** | **Both Counselling and mass media** |
| --- | --- | --- | --- |
| **Poorest** | Ref | Ref | Ref |
| **Poorer** | 1.84*** | 0.94*** | 1.49*** |
| **Middle** | 2.46*** | 0.92*** | 1.77*** |
| **Richer** | 3.64*** | 0.77*** | 2.36*** |
| **Richest** | 6.18*** | 0.70*** | 3.63*** |

* P value ≤ 0.05, ** P value ≤ 0.01, *** P value ≤ 0.001
