# Supplementary material for: Impact of family planning messages on modern contraceptive use: an observational study of women of reproductive age in Zambia 2007–2018
Source: BMJ Glob Health. 2026 Oct 30;11(10):e024080. doi: 10.1136/bmjgh-2026-024080 (PMC13723780; doi:10.1136/bmjgh-2026-024080)
Supplement: Supplementary file 3 [file bmjgh-11-10-s003.docx]

**Supplementary material 3:** ATE of receiving family planning messages through the different methods across wealth quintiles.

| **Wealth quintile** | **Mass media** | **Counselling** | **Both Counselling and mass media** |
| --- | --- | --- | --- |
| **Poorest** | 0.018 | 0.191*** | 0.224*** |
| **Poorer** | 0.070** | 0.133*** | 0.165*** |
| **Middle** | 0.027* | 0.128*** | 0.177*** |
| **Richer** | 0.045* | 0.148*** | 0.146*** |
| **Richest** | -0.001 | 0.068 | 0.104*** |

* P value ≤ 0.05, ** P value ≤ 0.01, *** P value ≤ 0.001
