# Supplementary material for: Impact of family planning messages on modern contraceptive use: an observational study of women of reproductive age in Zambia 2007–2018
Source: BMJ Glob Health. 2026 Oct 30;11(10):e024080. doi: 10.1136/bmjgh-2026-024080 (PMC13723780; doi:10.1136/bmjgh-2026-024080)
Supplement: Supplementary file 4 [file bmjgh-11-10-s004.docx]

**Supplementary material 4**

Supplement 1: Logistic regression of modes of delivery of family planning messages and modern contraceptive use (N=19,958)

| Mode of delivery of FP messages | Unadjusted models | | | | Adjusted models | | | |
| --- | --- | --- | --- | --- | --- | --- | --- | --- |
|  | **COR** | **Lower bound** | **Upper bound** | **P value** | **AOR** | **Lower bound** | **Upper bound** | **P value** |
| Mass media | 1.33*** | 1.21 | 1.46 | 0.000 | 1.18** | 1.06 | 1.31 | 0.002 |
| Counselling | 1.66*** | 1.50 | 1.84 | 0.000 | 1.47*** | 1.31 | 1.65 | 0.000 |
| Both Mass media & counselling | 2.19*** | 1.96 | 2.44 | 0.000 | 1.73*** | 1.55 | 1.94 | 0.000 |

*95% confidence interval; * P value ≤ 0.05, ** P value ≤ 0.01, *** P value ≤ 0.001*

*COR– Crude Odds Ratios*

*AOR– Adjusted Odds Ratios*
