# Supplementary material for: Impact of family planning messages on modern contraceptive use: an observational study of women of reproductive age in Zambia 2007–2018
Source: BMJ Glob Health. 2026 Oct 30;11(10):e024080. doi: 10.1136/bmjgh-2026-024080 (PMC13723780; doi:10.1136/bmjgh-2026-024080)
Supplement: Supplementary file 5 [file bmjgh-11-10-s005.docx]

**Supplementary material 5**

Bootstrapping results for AIPW with100 iterations

| Method of expsure to FP messages | ATE | Bootstrap std. err. | P value | Lower bound | Upper bound |
| --- | --- | --- | --- | --- | --- |
| Mass media | 0.0337 | 0.0104 | 0.001 | 0.0132 | 0.0542 |
| Counselling | 0.1455 | 0.0093 | 0.000 | 0.1273 | 0.1638 |
| Both mass media & counselling | 0.1706 | 0.0126 | 0.000 | 0.1458 | 0.1953 |

FP – family planning; 95% confidence interval
