# Supplementary material for: Impact of family planning messages on modern contraceptive use: an observational study of women of reproductive age in Zambia 2007–2018
Source: BMJ Glob Health. 2026 Oct 30;11(10):e024080. doi: 10.1136/bmjgh-2026-024080 (PMC13723780; doi:10.1136/bmjgh-2026-024080)
Supplement: Supplementary file 6 [file bmjgh-11-10-s006.docx]

### Reflexivity Statement

Adapted from Morton, B., Vercueil, A., Masekela, R., Heinz, E., Reimer, L., Saleh, S., Kalinga, C., Seekles, M., Biccard, B., Chakaya, J., Abimbola, S., Obasi, A. and Oriyo, N. (2022), Consensus statement on measures to promote equitable authorship in the publication of research from international partnerships. Anaesthesia, 77: 264-276. <https://doi.org/10.1111/anae.15597>

| **Study conceptualisation** | |
| --- | --- |
| 1. How does this study address local research and policy priorities? | Zambia envisages to increase the use of contraception from 48% as of 2018 to a national target of 70% among sexually active women by 2026. This is seen as one of the ways of making progress towards reducing unintended pregnancies and reducing teenage pregnancy from 29.2% to 25% as part of its Integrated Family Planning Costed Implementation Plan (2021-2026). Family planning messages are one of the interventions being used to make progress towards the said targets. Therefore, investigating the impact of family planning messages is essential as such evidence is relevant for informing policy. |
| 2. How were local researchers involved in study design? | The study was designed jointly with the local researchers including line of investigation and identification of relevant data. |
| **Research management** | |
| 3. How has funding been used to support the local research team(s)? | This study did not receive specific funding; however, it is part of JK’s PhD which is partly funded. As part of that funding, JK and FM were facilitated to travel between Sweden and Zambia to execute the study. |
| **Data acquisition and analysis** | |
| 4. How are research staff who conducted data collection acknowledged? | This is not applicable as the data used in the second is anonymised secondary data (ZDHS) whose source is acknowledged in the manuscript. |
| 5. How have members of the research partnership been provided with access to study data? | All research team members had access to the data stored on a secure university cloud storage platform. |
| 6. How were data used to develop analytical skills within the partnership? | The team members jointly discussed the methods and analytical approach |
| **Data interpretation** | |
| 7. How have research partners collaborated in interpreting study data? | The interpretation of the results was discussed among the team and agreed to by all co-authors |
| **Drafting and revising for intellectual content** | |
| 8. How were research partners supported to develop writing skills? | The research partners were involved in the writing and reviewing of the manuscript |
| 9. How will research products be shared to address local needs? | The research manuscript will be shared with the ministry of health officials in Zambia which is responsible for the contraceptive policy in the country. |
| **Authorship** | |
| 10. How is the leadership, contribution and ownership of this work by LMIC researchers recognised within the authorship? | The JK, the first author and FM are from LMIC. The first author led the study supervised by FM, JS and BE. |
| 11. How have early career researchers across the partnership been included within the authorship team? | JK is an early career researcher who led the study. |
| 12. How has gender balance been addressed within the authorship? | The co-authorship included both women and men. |
| **Training** | |
| 13. How has the project contributed to training of LMIC researchers? | This study is part of JK’s PhD studies who comes from LMIC. |
| **Infrastructure** | |
| 14. How has the project contributed to improvements in local infrastructure? | All team members got access to the data and analysis software. |
| **Governance** | |
| 15. What safeguarding procedures were used to protect local study participants and researchers? | Some of the safeguards put in place was respectful, regular and open communication among the coauthors where each member was free to air out their ideas, suggestions and options. |
